# Potent Anti-Inflammatory Activity of Novel Microtubule-Modulating Brominated Noscapine Analogs

**DOI:** 10.1371/journal.pone.0009165

**Published:** 2010-02-11

**Authors:** Susu Zughaier, Prasanthi Karna, David Stephens, Ritu Aneja

**Affiliations:** 1 Division of Infectious Diseases, Emory University School of Medicine, Atlanta, Georgia, United States of America; 2 Department of Biology, Georgia State University, Atlanta, Georgia, United States of America; BMSI-A*STAR, Singapore

## Abstract

Noscapine, a plant-derived, non-toxic, over-the-counter antitussive alkaloid has tubulin-binding properties. Based upon the structural resemblance of noscapine to colchicine, a tubulin-binding anti-inflammatory drug, noscapine and its semi-synthetic brominated analogs were examined for *in vitro* anti-inflammatory activity. Brominated noscapine analogs were found to inhibit cytokine and chemokine release from macrophage cell lines but did not affect cell viability. Brominated noscapine analogs demonstrated anti-inflammatory properties in both TLR- and non-TLR induced *in vitro* innate immune pathway inflammation models, mimicking septic and sterile infection respectively. In addition, electron microscopy and immunoblotting data indicated that these analogs induced robust autophagy in human macrophages. This study is the first report to identify brominated noscapines as innate immune pathway anti-inflammatory molecules.

## Introduction

The innate immune system plays a crucial role in host defense and homeostasis. Innate immune responses are induced when pattern recognition receptors such as Toll-like receptors (TLR) sense the presence of pathogen associated molecules (PAMPS) or endogenous “danger” molecules (DAMPS) released by damaged cells [1], [2]. Although acute innate pathway inflammatory responses promote termination of infection and wound healing, chronic activation of this pathway can lead to tissue damage and fibrosis and contribute to various disease states such as atherosclerosis, arthritis, inflammatory bowel disease and even cancer [1], [2], [3].

There is a need for new non-toxic agents that can treat inflammation. One class of anti-inflammatory agents is microtubule-disrupting agents such as colchicine and vinblastine. These agents have been shown to reduce TNFα production in macrophages due to their ability to impair tubulin dynamics [3]. In particular, colchicine, a robust microtubule-depolymerizing agent, has been widely employed for gout management. However, toxicities limit colchicine's usefulness [4]. Another tubulin inhibitor, vinblastine, used in the treatment of hematological malignancies, has significant toxic side-effects such as leucocytopenias, gastrointestinal toxicity, peripheral neuropathy and immunosuppression [5]. Thus, the search for non-toxic microtubule-binding agents that have anti-inflammatory activity continues.

**Figure 1:**
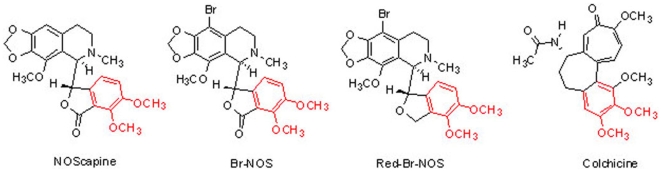
Molecular structures of noscapine, 9-bromonoscapine (Br-nos), reduced 9-bromonoscapine (Red-Br-nos) and colchicine. The common dimethoxy phenyl group is shown in red.

Noscapinoids constitute an emerging class of small-molecule microtubule-modulating agents that do not alter the total polymer mass of tubulin [6], [7], [8], [9], [10]. The parent molecule, noscapine, a plant-derived antitussive alkaloid has been recently found to have anticancer activity [11], [12], [13]. Rational drug-design and chemical-synthesis of potent noscapine analogs (noscapinoids) that retain the non-toxic attributes of the parent noscapine and are more efficacious in cancer cells has been reported [7], [8], [9], [10]. Particularly, brominated noscapine analogs [*(S)-3-((R)-9-bromo-4-methoxy-6-methyl-5,6,7,8-tetrahydro-* [*1*], [*3*] *dioxolo[4,5-g]isoquinolin-5-yl)-6,7-dimethoxyiso-benzofuran-1(3H)-one*, referred to as 9-bromonoscapine or Br-nos] and [*(R)-9-bromo-5-((S)-4,5-dimethoxy-1,3-dihydroisobenzofuran-1-yl)-4-methoxy-6-methyl-5,6,7,8-tetrahydro-* [*1*], [*3*] *di-oxolo-[4,5-g]isoquinoline*, referred to as reduced-9-bromonoscapine or Red-Br-nos] have been shown to have significant anticancer activity and are non-toxic at doses as high as 300 mg/kg [9], [10], [14], [15]. Perhaps due to limited effects on microtubule dynamics, noscapinoids do not perturb the transport functions of microtubules in a variety of cell types such as post-mitotic neurons and have no apparent histo-, hemato-, immuno- or neuronal toxicity [8], [9], [10], [14], [15], [16]. Another unique property of noscapinoids is their oral bioavailability [17]. Interestingly, a close look at molecular structure reveals structural resemblance of noscapine to colchicine, in that both have a common dimethoxy phenyl group (Figure 1). Based upon this structural similarity, the aim of this study was to investigate innate immune pathway anti-inflammatory activity of noscapine and its brominated analogs *in vitro*. We describe the anti-inflammatory activity of brominated noscapine analogs, 9-bromonoscapine (Br-nos) and the cyclic ether brominated analog (Red-Br-nos) in *in vitro* models mimicking septic and sterile innate immune pathway induced inflammation. The data demonstrate that these drugs inhibit TNFα, IL-8 and nitric oxide release upon challenge with various TLR and non-TLR ligands. This is the first report to identify and define the innate immune pathway anti-inflammatory activity of noscapine and its brominated analogs.

## Results

### Brominated Noscapine Analogs Possess Potent Innate Anti-Inflammatory Activity

**Figure 2:**
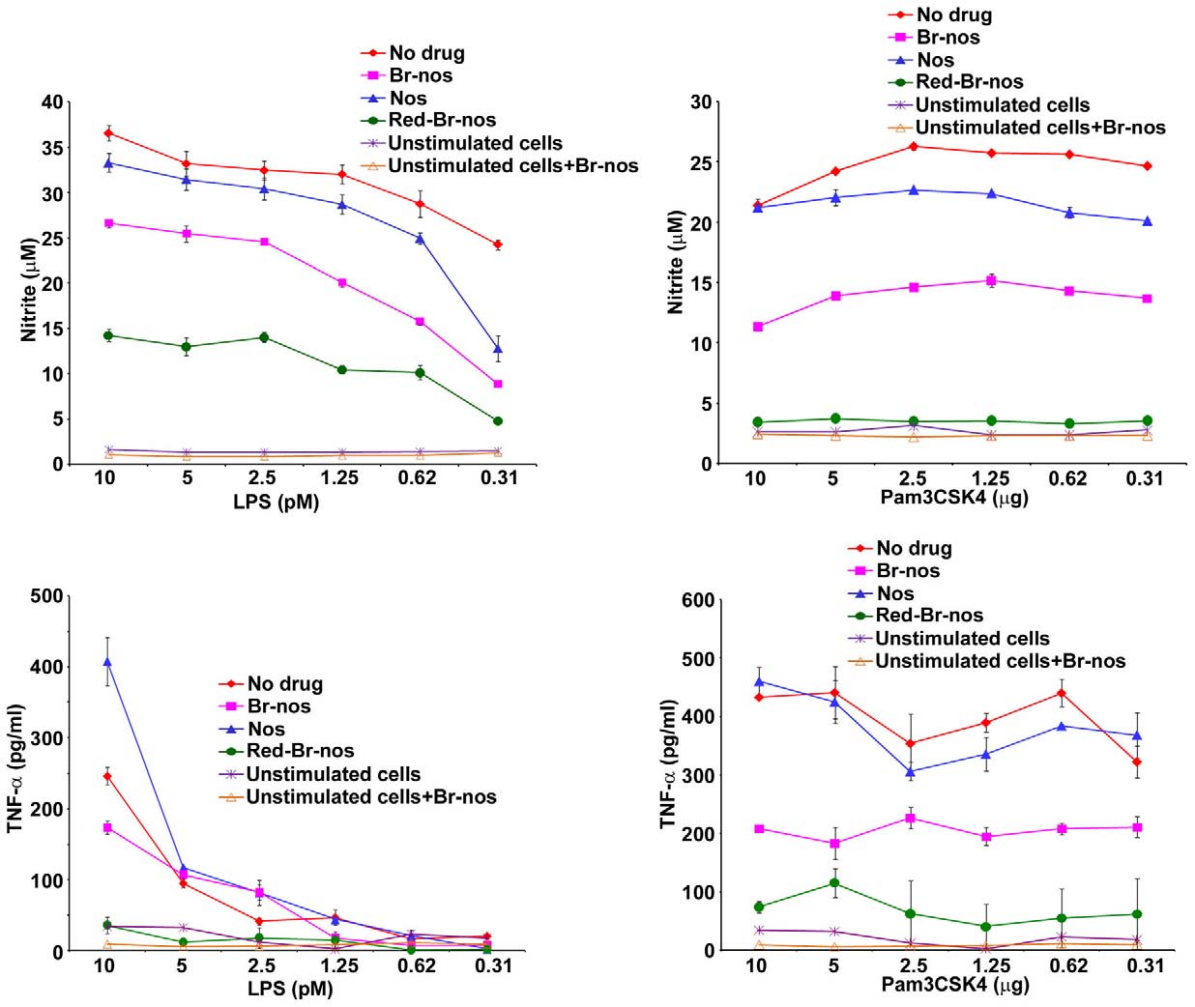
Pre-treatment of murine and human macrophages with brominated noscapine analogs significantly inhibit TLR4 and TLR2 pathway inflammatory responses. Murine RAW 264.7 and human THP-1 macrophages were treated with 50 µM of noscapine analogs for 4 hrs followed by LPS induction in concentrations ranging from 10-0.31 pM, or Pam3CSK4 in concentrations ranging from 10-0.31 µg/ml overnight. **A**- Nitric oxide (NO) release was determined as nitrite accumulation in supernatants detected by the Griess method. **B**- TNFα release into supernatants was measured by ELISA. **No drug**: RAW264.7 cells treated with DMSO (50 µM) alone followed by LPS or Pam3CSK4 induction. **Nos**: noscapine followed by the TLR ligand (nitric oxide *p* value for LPS = 0.0016, Pam3CSK4 = 0.00013 and TNFα *p* value for LPS = 0.002, Pam3CSK4 = 0.1). **Br-nos**: 9-bromonoscapine followed by the TLR ligand (*p* value<0.00001). **Red-Br-nos**: Reduced bromonoscapine and the TLR ligand (*p* value<0.0001). Unstimulated cells were used as control and these cells were treated either with DMSO only or Br-nos (50 µM) without the TLR ligand. Error bars represent SD from mean of at least duplicate readings. Data are representative of at least 3 independent experiments. *p* values were calculated with reference to no drug values.

**Figure 3:**
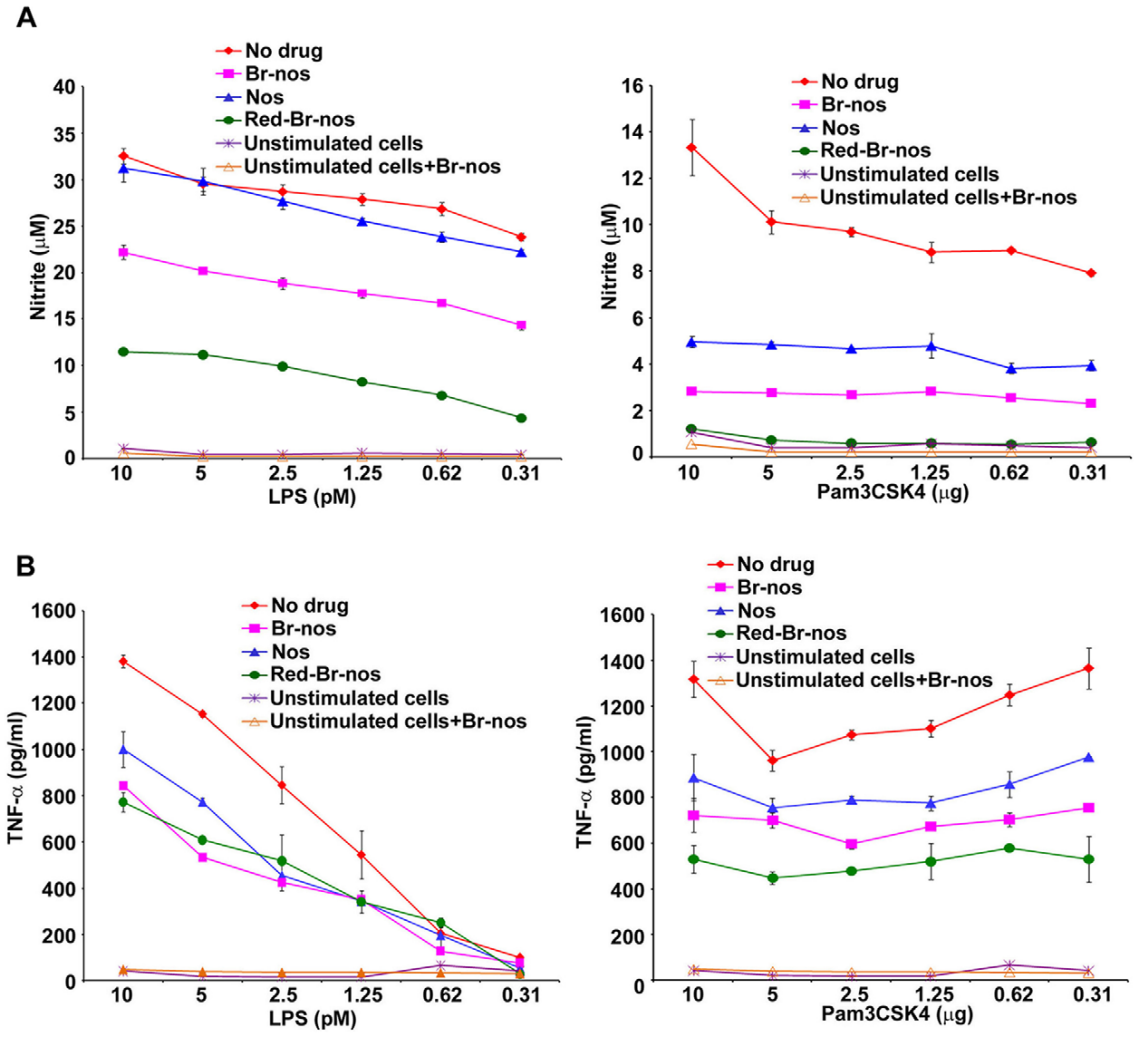
Co-treatment of murine and human macrophages with brominated noscapine analogs inhibit TLR-induced inflammatory responses. **A**: NO release from RAW 264.7 cells co-treated with 50 µM of noscapine and its analogs and varying doses of TLR ligands (LPS, TLR4 ligand or TLR2 ligand Pam3CSK4) overnight. Nitrite accumulation was detected by the Griess method. **B**: TNFα release from THP-1 human macrophage like cells co-treated with 50 µM of noscapine and its analogs and TLR ligands as above and incubated overnight. TNFα release was measured in the supernatants by ELISA. No drug: RAW264.7 cells treated with DMSO (50 µM) alone followed by LPS or Pam3CSK4 induction. For Nos (noscapine) and the TLR ligands, nitric oxide *p* value for LPS was = 0.03, Pam3CSK4 = 0.0004 and TNFα *p* value for LPS = 0.0002, Pam3CSK4 = 0.0004. For Br-nos (9-bromonoscapine) and the TLR ligands *p* values were <0.0001. For Red-Br-nos (Reduced bromonoscapine) and the TLR ligands *p* values were <0.00001. Unstimulated cells were used as control and these cells were treated either with DMSO only or Br-nos (50 µM) without the TLR ligand. Error bars represent SD from mean of at least duplicate readings. Data are representative of at least 3 independent experiments. *p* values were calculated with reference to no drug values.

**Figure 4:**
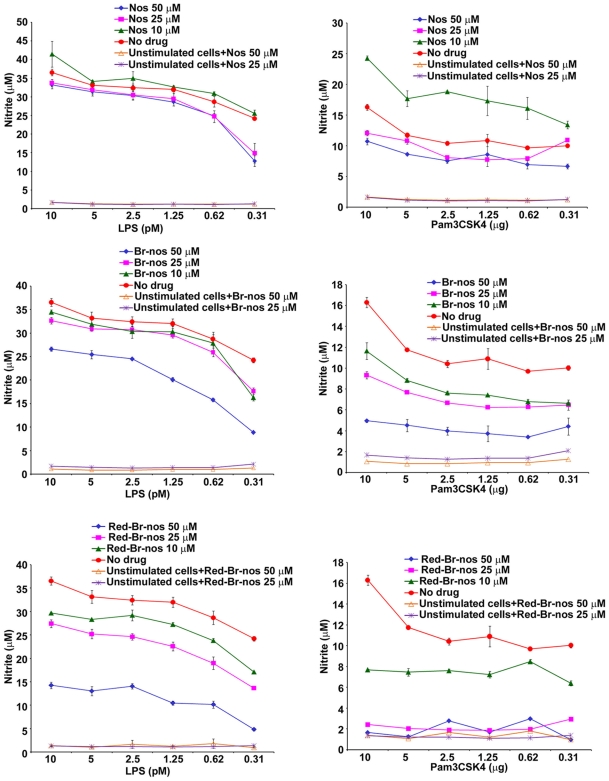
Dose-dependent anti-inflammatory effects of the brominated noscapine analogs. Nitric oxide release was measured in RAW 264.7 murine macrophages treated with 10, 25 and 50 µM of noscapine analogs for 4 hrs, then stimulated with the TLR4 ligand LPS or the TLR2 ligand Pam3CSK4 and incubated overnight. Nitrite accumulation was detected by the Griess method. Nos: noscapine parent analog (10 µM Nos concentration, *p* value for LPS = 0.014, Pam3CSK4 = 0.0007; 25 µM Nos *p* value for LPS = 0.0001, Pam3CSK4 = 0.00001; 50 µM Nos *p* values<0.00001). Br-nos: brominated noscapine analog and TLR ligand (10 µM Br-nos *p* value for LPS = 0.001, Pam3CSK4 p<0.00001; 25 µM Br-nos *p* value for LPS = 0.0003, Pam3CSK4, p value<0.00001; 50 µM Br-nos *p* value<0.00001). Red-Br-nos: reduced brominated noscapine and TLR ligand (10, 25 and 50 µM Red-Br-nos *p* value<0.00001). Unstimulated cells were used as control and were treated with 25 and 50 µM of noscapine analogs. Error bars represent the SD from the mean of at least duplicate readings. These data is representative of 3 independent experiments. *p* values were calculated with reference to no drug values.

Noscapine and brominated noscapine analogs (Br-nos and Red-Br-nos) were first investigated in a TLR ligand induced septic inflammation model. Human (THP-1) and murine (RAW 264.7) macrophages were used that usually respond well to a diverse repertoire of pathogens and inflammatory mediators. Macrophages were treated with noscapine or brominated noscapine analogs at 10, 25 and 50 µM prior to stimulation or co-stimulation with highly purified TLR-ligands (meningococcal LPS, a TLR4-MD-2 ligand and the synthetic lipopetide Pam3CSK4, a TLR2 ligand). Pre-treatment of murine RAW 264.7 and human THP-1 macrophages with brominated noscapine analogs (50 µM) for 4 hr followed by a 12 hr stimulation with the TLR4-MD-2 ligand meningococcal LPS (doses 10-0.31 pM) resulted in a significant reduction in nitric oxide and TNFα release compared to cells that were treated with DMSO (untreated control) and stimulated with LPS or Pam3CSK4 (Figure 2). Similar reduction in nitric oxide and TNFα release was observed in time-course of drug pre-treatment i.e. 1, 2 and 12 hrs (data not shown). Brominated noscapine analogs displayed significantly higher anti-inflammatory activity compared to the parent drug noscapine. Similarly, co-treatment of human THP-1 and murine RAW 264.7 macrophages with brominated noscapine analogs and TLR-ligands LPS and Pam3CSK4 for 12 hr resulted in significant reduction of TNFα and nitric oxide release (Figure 3). However, no effects on cell viability as determined using trypan blue vital dye exclusion, microscopic morphology and LDH release were observed in these studies. THP-1 cells incubated for 48 hr with 50 µM of either DMSO, Nos, Br-nos or Rd-Br-nos released 5, 7, 8 and 10 IU/L of LDH, respectively. Noscapine or brominated noscapine analogs alone did not induce inflammatory responses or impair cellular viability in human or murine macrophages. The brominated noscapine analogs dampened TLR-mediated TNFα and nitric oxide (NO) release from human and murine macrophages in a dose-dependent manner (Figure 4). In summary, these data demonstrated significant innate immune anti-inflammatory activity of brominated noscapine analogs to TLR ligands compared to untreated controls or the parent drug noscapine.

### Anti-Inflammatory Potential of Brominated Noscapines in Sterile Inflammation

**Figure 5:**
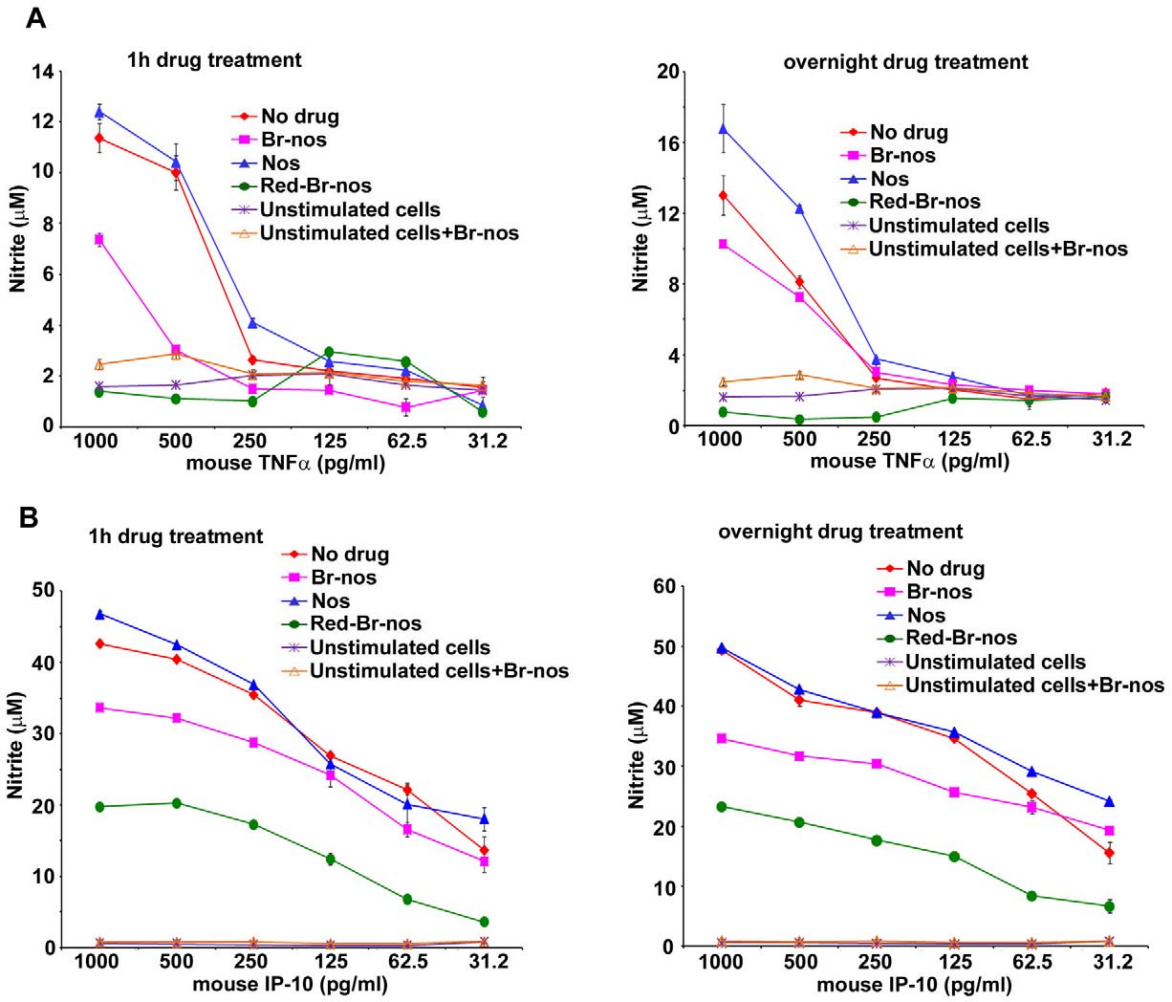
Brominated noscapine analogs dampen sterile inflammation. **A**: ScCr (TLR4-deficient) and **B**: RAW264 (TLR4-sufficient) cells were treated with 50 µM noscapine analogs for 1 hr (left panel) or overnight (right panel) then stimulated with the murine chemokine IP-10 (CXCL10) or the murine cytokine TNFα in concentrations from 1000-31.2 pg/ml and incubated overnight. NO release was measured in supernatants by the Griess method. No drug: ScCr cells treated with DMSO and stimulated with murine cytokine TNFα (**A**); RAW 264.7 cells stimulated with murine chemokine IP-10 (**B**). Nos: noscapine and non-TLR ligands TNFα (**A**) or IP-10 (**B**). Br-nos: 9-bromonoscapine and non-TLR ligands. Red-Br-no**s**: Reduced bromonoscapine and non-TLR ligands. Unstimulated cells were used as control and were treated either with DMSO only or Br-nos (50 µM). Error bars represent SD from mean of at least duplicate readings. *p* values were calculated with reference to no drug values and were <0.0001 for Br-nos and Red-Br-nos drug treatment and not significant for the parent drug noscapine.

The anti-inflammatory role of brominated noscapines was also confirmed in a sterile inflammation model mimicked by macrophages stimulated with recombinant cytokines and chemokines instead of TLR ligands. Human and murine macrophages as well as HEK-TLR transfected cells were induced with non-TLR ligands such as the cytokine TNFα or the chemokine IP-10/CXCL10. Brominated noscapine analogs significantly reduced nitric oxide release from murine macrophages ScCr (TLR4-deficient cells) stimulated with recombinant cytokine mouse TNFα (Figure 5A). Furthermore, brominated noscapine analogs significantly decreased nitric oxide release from murine RAW 264.7 (TLR4-sufficient cells) macrophages induced with recombinant mouse chemokine IP-10/CXCL10 (Figure 5B).

### Brominated Noscapine Analogs Do Not Bind TLR-4-MD-2 Receptor

**Figure 6:**
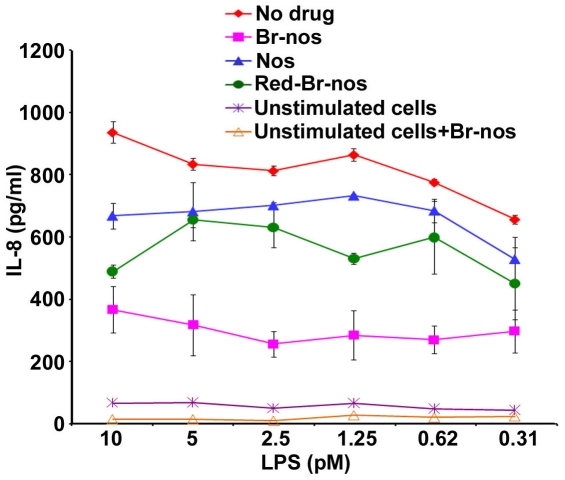
Brominated noscapine analogs dampen TLR-mediated IL-8 release. HEK293 cells stably transfected with human TLR4-MD-2-CD14 receptors were co-treated with 50 µM of noscapine analogs and LPS concentrations ranging from 10-0.31 pM and incubated overnight. IL-8 release in supernatants was determined by ELISA. No drug: RAW264.7 cells treated with DMSO (50 µM) alone followed by LPS or Pam3CSK4 induction. Nos: noscapine and the TLR ligand. Br-nos: 9-bromonoscapine and the TLR ligand. Red-Br-nos: Reduced bromonoscapine and the TLR ligand. Unstimulated cells were used as control and were treated either with DMSO only or Br-nos (50 µM) without the TLR ligand. *p* values were calculated with reference to no drug values and were <0.0001 for Nos, Br-nos and Red-Br-nos drug treatment.

To confirm the anti-inflammatory effects of brominated noscapine analogs, HEK293 human epithelial cells stably-transfected with TLR4-MD-2-CD14 receptors were co-treated with noscapine analogs (50 µM) and varying LPS concentration followed by overnight incubation. Brominated noscapine analogs significantly reduced TLR4-MD-2 mediated IL-8 release from HEK/TLR4-MD-2-CD14 cells (Figure 6). These drugs also significantly reduced IL-8 release by ∼3 fold (no drug = 1400 pg/ml, Br-nos = 650 pg/ml, Red-Br-nos = 400 pg/ml) from HEK293 cells transfected with TLR2 alone or TLR2/6 receptors induced with Pam3CSK4. Brominated noscapine analogs decreased inflammatory responses induced by both LPS (a TLR4-MD-2 ligand) and by Pam3CSK4 (a TLR2 ligand) suggesting that brominated noscapine analogs did not exert their anti-inflammatory effect by selectively inhibiting TLR4 and TLR2 cell surface receptor dimerization. Unlike paclitaxel that binds human and murine MD-2 [24], the anti-inflammatory role of brominated noscapines appeared to be due to effects on tubulin dynamics.

### Red-Br-nos Induces Robust Autophagy in Human Macrophage Cells

**Figure 7:**
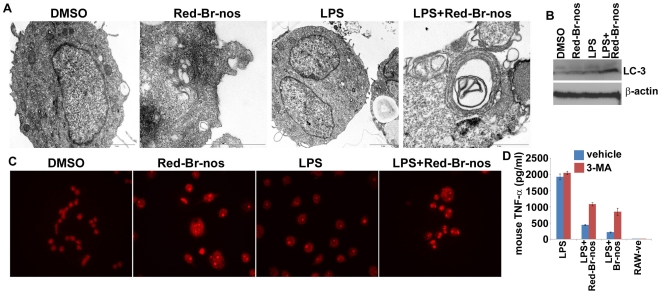
Brominated noscapine analogs induce autophagy in human macrophages. **A**: Representative electron micrographs of human macrophage cells THP-1 treated overnight with LPS alone (2 pM concentration) or LPS and reduced bromo-noscapine (50 µM). Drug induced autophagy was noted with increased vacuolation in the cytoplasm. **B**: Western blot of autophagy marker LC3 protein in cell pellets shown in panel A. **C**: Fluorescence microscopic analysis of THP-1 cells treated for 24 hrs with DMSO (control), Red-Br-nos, LPS, Red-Br-nos and LPS followed by staining with 1 µg/ml acridine. Note the formation (red fluorescence) of acridine orange–accumulating acidic vesicular organelles (AVOs) in Red-Br-nos treated cells. **D**: Mouse TNFα release from RAW264.7 cells treated with the autophagy inhibitor 3-MA (50 µM) prior to stimulation with LPS alone or with 50 µM of Red-Br-nos, Br-nos or Nos. TNFα was measured by ELISA. The data represent two independent experiments. *p* values were calculated with reference to no 3-MA inhibitor and were <0.001 for Nos, Br-nos and Red-Br-nos drug treatment and LPS stimulation.

The brominated noscapine analogs displayed innate anti-inflammatory activity with no effect on cellular viability. However, the underlying mechanism of the exerted anti-inflammatory activity is not known. Autophagy plays an essential role in cellular homeostasis and host defense and it is becoming evident that TLRs are the environmental sensors for autophagy associated with innate immunity [25], [26], [27]. Thus, drug-induced autophagy was investigated as a possible underlying mechanism of the anti-inflammatory effects of brominated noscapine analogs. Electron microscopy data showed that brominated noscapine analogs induced extensive autophagy-related vacuolation in human macrophages THP-1 (Figure 7A). This drug-induced autophagy was also confirmed by immunoblotting for microtubule-associated protein light chain 3 (LC-3), a well known marker for autophagy [28], [29]. The results demonstrate the conversion of LC3-I, the cytoplasmic form of LC-3, into LC3-II that incorporates into the autophagic membrane (Figure 7B). Detection of acidic vesicular organelles (AVOs) in THP-1 cells treated with Red-Br-nos further confirmed the drug-induced autophagic activity (Figure 7C). Further, when autophagy was inhibited by the pharmacological inhibitor 3-MA, a reduction in anti-inflammatory activity exerted by brominated noscapine analogs was observed. Murine RAW264.7 macrophages treated with 3-MA prior to stimulation with LPS and brominated noscapine analogs released more TNFα compared to macrophages without the autophagy inhibitor, 3-MA (Figure 7D). These data suggest that autophagic clearance mechanisms might be responsible for either dissipating the cytosolic inflammatory signaling complex before it reaches the nucleus to induce pro-inflammatory gene expression or might slow down/decrease protein synthesis leading to reduced release of pro-inflammatory mediators [30].

### Brominated Noscapine Analogs Enhance ROS Release in Macrophages

**Figure 8:**
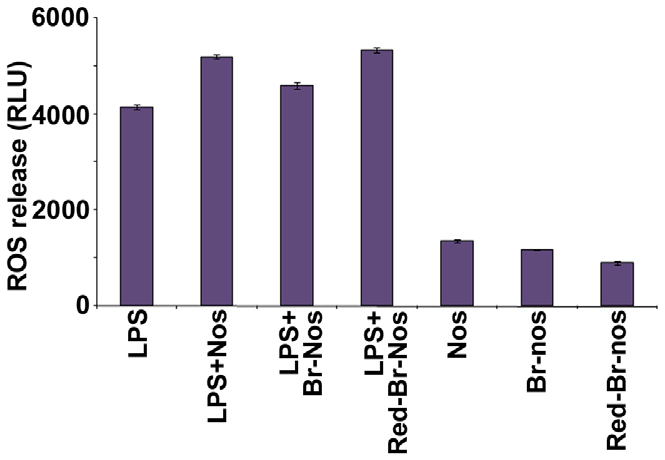
Noscapine analogs enhanced ROS release. ROS release from THP-1 macrophages primed with LPS (1 pM) overnight. Noscapine analogs (50 µM) were added just prior to a respiratory burst triggered with PMA (1 µM). ROS release was detected with the chemiluminescent probe lucigenin. *p* values were calculated with reference to no drug but LPS treated cells and were <0.002.

Oxidative and respiratory burst are vital cellular functions that play an important role in homeostasis and host defense. Oxidative burst leads to the release of highly reactive oxygen species (ROS) radicals. We have previously shown that brominated noscapine analog induces a mitochondrially-driven intrinsic apoptotic cascade by dissipation of the mitochondrial membrane potential [9], [10]. Since changes in mitochondrial membrane potential are usually associated with increases in ROS release [31], the effects of brominated noscapine analogs on ROS release from LPS-primed human macrophages were investigated using an enhanced chemiluminescence method [22]. The results showed that both noscapine and its brominated analogs enhanced ROS release in LPS primed cells when added just prior to triggering the respiratory burst, but not in unprimed macrophages (Figure 8). In contrast, no change in ROS release was seen when noscapine or its analogs were added to primed macrophages 2 hr prior to triggering the respiratory burst (data not shown). The data suggest that the enhanced ROS release from primed macrophages may perhaps be due to effects of the drug on mitochondrial membrane potential rather than cellular signaling and macrophage priming.

## Discussion

Noscapinoids bear structural similarity to colchicine, in that they have a common molecular signature of a dimethoxy phenyl group (Fig. 1, see groups in red). This study indicates that brominated noscapine analogs have potent anti-inflammatory activity in septic and sterile inflammation models, in a dose-dependent and time-dependent manner. Brominated noscapine analogs (Br-nos and Red-Br-nos) dampened TLR-mediated TNF-α and nitric oxide (NO) release in human and murine macrophages without evidence of cellular toxicity. Brominated noscapine analogs also inhibited cytokine/chemokine (a non-TLR ligand) induced inflammation that mimics sterile inflammation. These data suggest that the anti-inflammatory activity of brominated noscapine analogs is not due to a direct inhibition of TLR-receptor dimerization and signal initiation.

Two major classes of tubulin-binding drugs, namely *taxanes* (such as taxol, taxotere) and *vincas* (vinblastine, vincristine) are extensively studied. While *taxanes* overpolymerize and bundle microtubules into sheets, *vinca* alkaloids depolymerize microtubules into soluble tubulin. Owing to their extreme effects on microtubules, both these classes of drugs interfere with microtubular-track dependent trafficking and cause toxicities such as peripheral neuropathies, gastrointestinal toxicity and immunosuppression [5]. Unlike taxanes and vincas, noscapine and its analogs subtly attenuate microtubule dynamics and do not alter the monomer/polymer ratio of tubulin[6], [13], which perhaps is the reason for their non-toxic properties [7], [8], [9], [10], [11], [12], [13], [14], [15], [16], [32]. Recently, we have shown by detailed quantitative analysis that brominated noscapine (EM011) modulates microtubule dynamics by increasing the ‘pause’ time of microtubules without affecting their overall existence [32]. Thus, noscapine and its brominated noscapine analog has been shown to be non-toxic with ‘kinder and gentler’ effects on microtubules [33]. Interestingly, they possess potent anti-inflammatory activity and drug-induced attenuation of microtubule dynamics might provide a possible mechanism contributing to dissipating cytosolic signaling thus dampening inflammatory responses.

Brominated noscapine analogs when added to macrophages significantly induced autophagy as evident by extensive autophagy-related vacuolation in cytoplasm and upregulation of microtubule-associated light-chain protein, LC3-II, that integrates into the autophagic vesicles. Further, the inhibition of autophagy with 3-MA led to reduced anti-inflammatory activity exerted by brominated noscapine analogs. Thus, these data suggest that induction of autophagy is in part mediating the anti-inflammatory activity of noscapine analogs. Autophagy is a well-recognized ancient conserved cellular pathway that removes macromolecules, recycles and degrades unwanted cytoplasmic components [30]. The autophagosome fuses with the lytic lysosome to form autophagolysosomes that degrades engulfed molecules [34]. Recently, the role of autophagy in host defense has become evident. TLRs are the environmental sensors for autophagy associated with innate immunity [25], [26]. Several recent reports demonstrate that autophagy, a bulk degradation system, is directly involved in the control of inflammatory immune responses [30]. Genetic deficiency of Atg16L1, an autophagy protein, results in amplified inflammatory responses upon TLR stimulation as demonstrated by increased IL-1β release [30]. Thus, an essential component of the autophagic machinery, Atg16L1, suppresses endotoxin-induced intestinal inflammation [30]. Given the significant induction of autophagy by brominated noscapines, it is reasonable to speculate that autophagy might play a role in preventing or controlling inflammation. Thus, the cross-talk between drug-induced and TLR-mediated autophagic responses might impact inflammatory responses.

Oxidative and respiratory burst are vital cellular functions that play an important role in homeostasis and host defense. Oxidative burst leads to the release of highly reactive oxygen species (ROS) radicals. Respiratory burst can be triggered by phagocytosis, drugs and toxins as well as by soluble stimulus like the protein kinase C activator PMA. ROS contribute to host defense by killing the invading pathogen and also act as a second messenger that induce release of chemokines and cytokines. We have previously reported that host cationic peptides inhibit cytokine release from macrophages primed with endotoxin but enhanced ROS release [21]. Although the underlying mechanism for ROS amplification is not clear, these peptides seem to exert catalytic effect on NADPH oxidases, xanthine oxidase and cytochrome c. The current study shows that noscapine and its brominated analogs enhanced ROS release in LPS primed human macrophage cells but not in unprimed macrophages when added just prior to triggering the respiratory burst. Since noscapinoids are known to influence mitochondrial membrane potential [9], [14], the increase in ROS release may be due to the decline of mitochondrial transmembrane potential. One of the triggers for mitochondrial mediated apoptosis is the production of ROS. Recently, several studies have indicated that ROS also play a role in induction of autophagy [35]. The functional relationship between apoptosis (‘self-killing’) and autophagy (‘self-eating’) is intricate and complex in the sense that, under certain situations, autophagy constitutes a stress adaptation that avoids cell death (and suppresses apoptosis), whereas in other extreme cellular scenarios, it represents an alternative cell-death pathway [36].

Several reports suggest that unlike conventional tubulin-binding agents, noscapine and its analogs are devoid of any detectable toxicity to normal cells [8], [9], [10], [11], [12], [13], [14], [15], [16], [32]. Although the time-course employed to study inflammatory responses in macrophages (up to 24 hrs) has shown to preserve cell viability and no toxicity was observable, the long term effect of noscapine and its analogs on macrophages is under current investigation in our laboratory. We are also addressing whether brominated noscapine analogs differentially modulate genes involved in inflammation using gene expression profiles.

**Figure 9:**
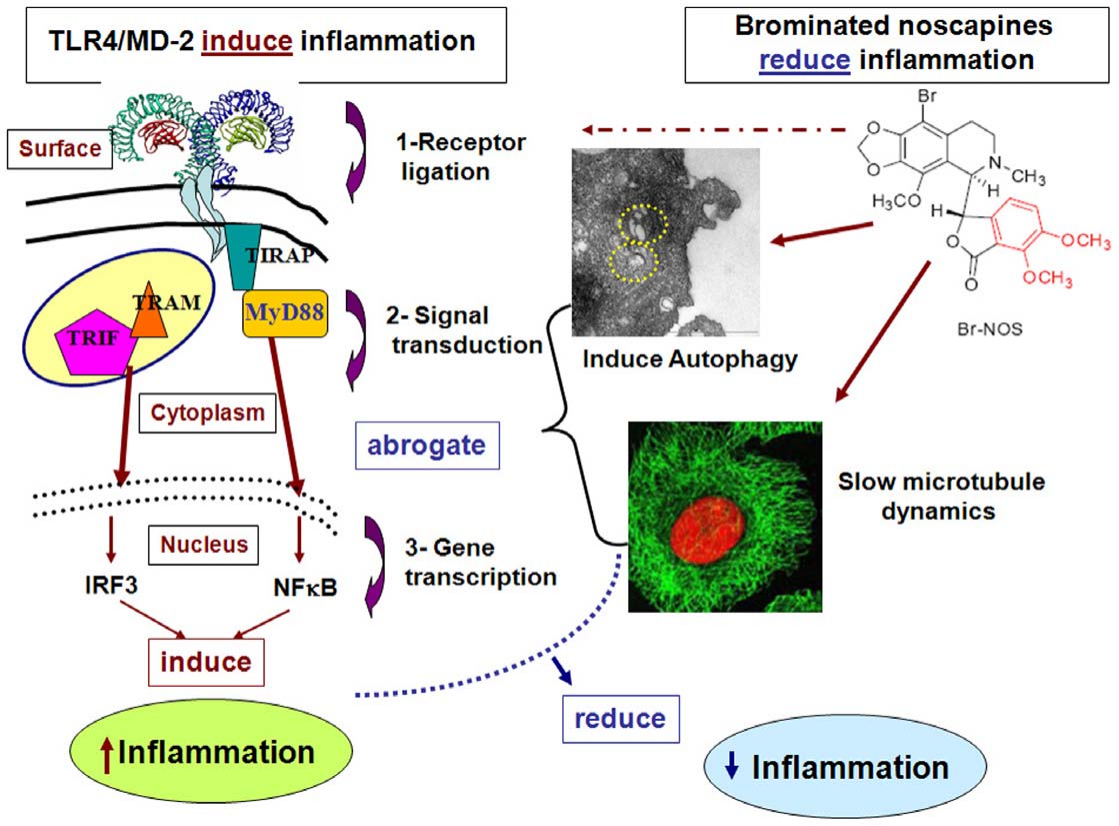
Schematic presentation of TLR4/MD-2 induced inflammation and hypothetical mode of action for the drug induced anti-inflammatory effect. Brominated noscapine analogs exert anti-inflammatory effect by interfering with targets either at **1**-cell surface receptor ligation, **2**- abrogating signal transduction by attenuating microtubule dynamics and/or by inducing autophagy, **3**-inhibiting gene transcription in the nucleus. TLR4/MD-2 signaling model is adapted from Park et al., 2009 [36] and the embedded images are from the current study.

In summary, brominated noscapine analogs display innate anti-inflammatory activity without affecting cell viability. Our data suggest that non-toxic brominated noscapine analogs perhaps inhibit the pro-inflammatory responses by inducing autophagy that dampens inflammation by attenuating or recycling the inflammatory signaling complex. Brominated noscapine analogs attenuate microtubule dynamics without altering the monomer/polymer ratio of tubulin [6], [32]. Thus, the anti-inflammatory effect of these non-toxic brominated noscapine analogs may also be due to a direct affect on tubulin and the associated slow dynamics might consequently dampen signal transduction or delay/impede protein transcription. Our hypothetical mechanism for anti-inflammatory mode of action of the brominated noscapine analogs is illustrated in a schematic model (Figure 9). These novel drugs may serve as useful tools to study the involvement of the microtubular system in inflammatory signaling.

## Materials and Methods

### Reagents

Noscapine analogs were synthesized according to previously reported methods [6], [7] and dissolved in DMSO. RPMI 1640 medium, Dulbecco's Eagle medium, fetal bovine serum (FBS), penicillin/streptomycin, sodium pyruvate and nonessential amino acids were obtained from Cellgro (Mediatech Herndon, VA). Autophagy inhibitor 3-methyladenine (3-MA) was purchased from Sigma (St Louis, MO). Synthetic TLR2 ligand Pam3CSK4, embryonic kidney 293 HEK-TLR2/6, HEK-TLR2 and HEK-TLR4-MD2-CD14 stably transfected cells were purchased from InvivoGen (San Diego, CA). Macrophage cell lines THP-1, RAW264.7 and 23ScCr were purchased from ATCC.

### Cell Cultures

THP-1 human macrophage-like cells were grown in RPMI 1640 with L-glutamate supplemented with 10% FBS, 50 IU/ml of penicillin, 50 µg/ml of streptomycin, 1% sodium pyruvate and 1% non-essential amino acids. Culture flasks were incubated at 37°C with humidity under 5% CO_2_. Murine macrophages (RAW 264.7, 23ScCr) and human kidney epithelial cells HEK293 were grown in Dulbecco's Eagle medium supplemented and incubated as mentioned above.

### LPS Purification and Quantitation

Lipopolysaccharide (LPS) or endotoxin is a well-characterized TLR4-MD-2 ligand. Endotoxin from the serogroup B *Niesseria meningitidis* strain NMB was initially extracted from whole meningococci by the phenol-water method. The endotoxin preparations were further purified and quantified as described [18]. Briefly, residual membrane phospholipids were removed by repeated extraction of the dried LPS (also known as lipooligosaccharide or LOS) samples with 9∶1 ethanol∶water. The expected fatty acyl components of 3-OHC12∶0, 3-OHC14∶0 and C12∶0 and the absence of membrane phospholipids was assessed by mass spectroscopy (GC-MS) (Dr Russell Carlson, Complex Carbohydrate Research Center, University of Georgia, Athens, GA). Endotoxin stock solutions were prepared in pyrogen free water at 10 nmole/ml concentration and further diluted with endotoxin free PBS to 1 nmole/ml and 100 pmole/ml with extensive vortex and sonication prior to each dilution [18], [19].

### Cellular Activation and Inflammatory Responses

The effect of noscapine analogs was investigated in time-course and dose-response experiments in well established human and murine cell lines. Human THP-1 (a macrophage-like cell line), murine RAW 264.7 (TLR4-sufficient), 23ScCr (TLR4-deficient), HEK-TLR2/6 and HEK-TLR4-MD2-CD14 stably transfected cell lines were treated with noscapine or its analogs at 10, 25 and 50 µM for either 1, 2, 4 hr or overnight prior to stimulation with TLR ligands mimicking septic inflammation or non-TLR ligands mimicking sterile inflammation. Alternatively, cells were co-treated with noscapine or its analogs (50 µM) and TLR ligands or non-TLR ligand e.g. recombinant cytokines/chemokines were then incubated overnight. TLR ligand concentrations ranging from (LPS: 10-0.31 picomolar (pM) and Pam3CSK4: 10-0.31 µg/ml) and non-TLR ligands (mouse IP-10 and TNFα: 1000-31 pg/ml) were made in duplicate wells using sterile PBS by serial fold dilutions in the 96-well tissue culture plates at 50 µl final volumes. Freshly grown THP-1 cells, RAW264.7, ScCr, HEK-TLR2/6 and HEK-TLR4-MD2-CD14 transfected cells each adjusted to 10^6^ cell/ml and 250 µl aliquots were dispensed into each well at a final cell density of 250×10^3^ in the designated 96-well plates. The plates were then incubated overnight at 37°C with 5% CO_2_ and humidity. Supernatants from stimulated cells were harvested and stored at −20°C until further use. In certain experiments THP-1 and RAW264.7 cells were pretreated with 50 µM of 3-MA, the pharmacological inhibitor of autophagy, for 30 min prior to co-treatment with TLR ligand and brominated noscapine analogs.

### Cytokine and Chemokine Profiles

Released cytokines TNFα and IL-6 and chemokines CXCL10 (IP-10), MIP-2α and IL-8 were quantified by DuoSet ELISA (R&D Systems, Minneapolis, MN) following the manufacturer's instructions as previously described [19].

### Nitric Oxide Induction by Murine Macrophages

Freshly grown adherent RAW 246.7 or 23ScCr (TLR4-deficient) macrophages were scraped by a cell scraper. Harvested cells were washed and re-suspended in Dulbecco's complete media, counted and adjusted to 10^6^ cell/ml. 250 µl aliquots were then dispensed into each well at final 250×10^3^ cell density in the designated 96-well plates prior to stimulation with TLR ligands or recombinant cytokines as mentioned above. The induced RAW 264.7 or 23ScCr macrophages were incubated overnight at 37°C with 5% CO_2_ and supernatants were harvested and saved. Nitric oxide release was quantified using the Griess chemical method as previously described [18].

### Cellular Viability and Proliferation Assessment

The toxicity of noscapine or its analogs was determined by assessing cellular viability and proliferation using trypan blue exclusion method [20]. Cells were grown at a starting density of 0.75 million cell/ml (final volume 2 ml) in presence of increasing doses (10, 25 and 50 µM/10^6^ cells) of noscapine or its analogs for 3 days. Cellular aliquots (100 µl) were taken daily and cells were diluted 1∶1 with the vital dye trypan blue 0.4% solution (Cellgro, Mediatech Inc, Herndon, VA) in PBS and viable cells were counted. LDH release into the supernatant was also measured to assess the toxicity of the drugs. Light microscopy imaging was used to assess cellular morphology of macrophages incubated with or without brominated noscapine analogs [21].

### Cellular Respiratory Burst (Oxidative Burst) Activity

The enhanced chemiluminescence method was used as previously described [22]. Briefly, freshly grown THP-1 cells were adjusted to 2×10^6^/ml, transferred to two large tissue culture flasks labeled as primed or unstimulated. Two pmole/ml LPS were added to prime cells or left unstimulated as control cells. Both flasks were incubated overnight at 37°C under 5% CO_2_. Primed and control cells were then aliquoted into designated small tissue culture flasks (25 ml volume) and treated with 50 µM of noscapine or its analogs or DMSO for 2 hr or instantly prior to triggering oxidative burst. Unprimed or control cells were also treated with 50 µM of noscapine or its analogs and incubated in the same way but without endotoxin. The cells were collected in universal tubes, washed twice with culture medium and resuspended in standard buffer (4.58 mM KH_2_PO_4_, 8.03 mM NaHPO_4_, 0.5 mM MgCl_2_, 0.45 mM CaCl_2_, 1% (w/v) glucose, 0.033% (w/v) KCl, 0.76% (w/v) NaCl and 0.1% (w/v) endotoxin-free bovine serum albumin (pH 7.3) at 2×10^6^/ml. The chemiluminescence probe lucigenin (Sigma) was added to the cell suspension (25 µl/ml of cells from 1.0 mM stock solution) and mixed gently. Aliquots (150 µl) of the cellular mixture were transferred into at least quadruplicate wells of a white 96-well plate (FluoroNunc-PolySorp; Nalge Nunc International, Rochester, NY). The respiratory burst was triggered with 50 µl of PMA (1 µM). Chemiluminescence was measured in relative light units (a measure of the number of photons generated by the reaction at each time point). Chemiluminescence was measured using a luminometer (ML3000, Dynatech Laboratories Inc. Chantilly, Virginia) and the plate was read immediately and then at 2 min intervals for the next 90 min [22].

### Electron Microscopy

Human macrophage cells were fixed for 1 hr with 2% glutaraldehyde in 0.1 M sodium cacodylate buffer, rinsed in the same buffer and the post-fixed with 2% osmium tetroxide in the same buffer for 1 hr at room temperature. The samples were then dehydrated with a graded ethanol series through 3X 100%, 2X in propylene oxide and embedded in Spurr's resin. Sections were stained with 1% uranyl acetate and lead citrate. Electron microscopy imaging was then performed at a magnification of 3597X.

### Detection of Acidic Vesicular Organelles (AVOs)

THP-1 cells were plated on cover slips and allowed to attach. Following treatments for 24 hr with DMSO alone (control), Red-Br-nos, LPS, Red-Br-nos and LPS, cells were stained with 1 µg/ml acridine orange for 15 min, washed with PBS, and examined under a Zeiss fluorescence microscope using a 40X objective lens [23].

### Western Blot Analysis

Proteins from THP-1 cells pellets were resolved by polyacrylamide gel-electrophoresis and transferred onto polyvinylidene difluoride membranes (Millipore). The membranes were blocked in Tris-buffered saline containing 0.2% Tween-20 and 5% fat-free dry milk and incubated first with primary antibodies (LC3 and β-actin, Cell Signaling) and then with horseradish peroxidase-conjugated secondary antibodies (Santa-Cruz). Specific proteins were visualized with enhanced chemiluminescence detection reagent according to the manufacturer's instructions (Pierce Biotechnology).

### Statistical Analysis

Mean values of at least 4 independent determinations ± SD and *p* values (Student *t* test) were calculated in reference to no drug treatment values using the Excel software.
